# Contrasting influences of two dominant plants, *Dasiphora fruticosa* and *Ligularia virguarea*, on aboveground and belowground communities in an alpine meadow

**DOI:** 10.3389/fmicb.2023.1118789

**Published:** 2023-04-14

**Authors:** Hongxian Song, Ziyang Liu, Hanwen Cui, Jingwei Chen, Shuyan Chen, Haining Gao, Xiaoli Yang, Yajun Wang, Jiajia Wang, Kun Liu, Sa Xiao, Lizhe An, Uffe N. Nielsen

**Affiliations:** ^1^Ministry of Education Key Laboratory of Cell Activities and Stress Adaptations, School of Life Sciences, Lanzhou University, Lanzhou, Gansu, China; ^2^State Key Laboratory of Herbage Improvement and Grassland Agro-Ecosystems, College of Ecology, Lanzhou University, Lanzhou, Gansu, China; ^3^College of Life Sciences and Engineering, Hexi University, Zhangye, Gansu, China; ^4^Hawkesbury Institute for the Environment, Western Sydney University, Penrith, NSW, Australia

**Keywords:** allelopathy, facilitation, high-throughput sequencing, piecewise structural equation modelling, biotic factors, abiotic factors

## Abstract

Soil organisms are abundant, phylogenetically and functionally diverse, and interact to catalyse and regulate critical soil processes. Understanding what structures belowground communities is therefore fundamental to gaining insight into ecosystem functioning. Dominant plants have been shown to influence belowground communities both directly and indirectly through changes in abiotic and biotic factors. In a field study, we used piecewise structural equation modelling to disentangle and compare the effects of a dominant allelopathic plant, *Ligularia virgaurea*, and a dominant facilitative plant, *Dasiphora fruticosa*, on understory plant, soil microbial and nematode community composition in an alpine meadow on the Tibetan plateau. *Dasiphora fruticosa* was associated with changes in edaphic variables (total nitrogen, soil organic carbon, pH and ammonium), understory plant and soil bacterial communities, whereas *Ligularia virguarea* was associated with increased soil ammonium content and soil fungal richness relative to dominant plant-free control plots. Moreover, nematode richness was significantly greater under *D. fruticosa*, with no change in nematode community composition. By contrast, nematode richness under *Ligularia virgaurea* was similar to that of dominant plant-free control plots, but nematode community composition differed from the control. The effects of both plants were predominantly direct rather than mediated by indirect pathways despite the observed effects on understory plant communities, soil properties and microbial assemblages. Our results highlight the importance of plants in determining soil communities and provide new insight to disentangle the complex above- and belowground linkages.

## Introduction

Soil organisms play a major role in ecosystem functions (Neher, 1999; Bongiorno et al., 2019). Soil microbes govern essential processes including litter decomposition, carbon (C) and nutrient cycles, energy transforms, and hence plant growth (Miransari, 2013; Gougoulias et al., 2014; Krishna and Mohan, 2017). The larger soil fauna regulates microbial activities and contributes to soil functioning more broadly (Bardgett et al., 1999; Schuldt et al., 2018; Zhou et al., 2022). Nematodes are small semi-aquatic multicellular animals and the most abundant animals in terrestrial ecosystems (Bardgett and van der Putten, 2014; van den Hoogen et al., 2019). Nematode communities are very diverse in most soils, spanning multiple trophic levels from primary consumers to predators (Yeates et al., 1993; Lazarova et al., 2021). Nematodes occupy a central position in the soil food web linking primary producers and primary consumers with higher trophic levels (Yeates, 2010; Li et al., 2014; Wan et al., 2022). They influence plant productivity (Lafont et al., 2007), the growth, distribution and metabolic activities of microorganisms (Grewa and Wright, 1992; Ruess and Dighton, 1996) and microbial community composition (Djigal et al., 2004), thereby regulating the decomposition pathways (Hu et al., 2015), decomposition rate (De Mesel et al., 2003) and nutrient cycling (Sohlenius et al., 1988; Chen et al., 2019). Moreover, omnivores and predators feed on other soil biota, which can affect the community composition of soil biota communities more broadly (Laakso and Setälä, 1999).

There is increasing interest in the linkages between plants and belowground communities given the observed effects on ecosystem functioning (Abgrall et al., 2018; Shao et al., 2018; Wang et al., 2018). At large scales, factors such as climate and soil type play a significant role in shaping belowground communities (Stevnbak et al., 2012; Tsiafouli et al., 2017), whereas plant species diversity and composition (Johnson et al., 2003; Wardle et al., 2003; De Deyn et al., 2004) and edaphic properties (Fierer, 2017; Jia et al., 2020) moderate composition at smaller scales. Dominant plants are particularly important given their role as ecosystem engineers, influencing belowground communities through changes in resource inputs and *via* their effect on understory communities and edaphic properties (Hortal et al., 2015). More broadly, dominant plants influence the surrounding environment by buffering microclimates and providing protection from wind, ultraviolet radiation and large herbivores (Li et al., 2011; McIntire and Fajardo, 2014; Saixiyala et al., 2017). This can have ecosystem-wide effects on belowground communities and biochemical cycles.

Soil nematode communities are highly diverse both spatially and temporally (Pen-Mouratov et al., 2004) and have attracted much attention as bio-indicators (Bongers and Ferris, 1999; Neher, 2001). Nematode community composition is influenced by the spatiotemporal variability in vegetation, soil properties, and microbial communities (Neher, 1999; Kardol et al., 2010; Kerfahi et al., 2016; Wilschut et al., 2019). For example, changes in the aboveground plant community alter nematode community composition (Yeates et al., 1993). Wang et al. (2019) demonstrated that nematode biomass was related to soil nitrogen (N) content, soil carbon (C) content, ammonium and pH. A recent global study found that soil characteristics explain most of the variation in nematode abundances, with greater numbers in soils with high organic C content and lesser when soil pH is very low (van den Hoogen et al., 2019). Moreover, soil water content has a positive effect on nematode abundances at least where water availability is low (Ruan et al., 2012). Dominant plants are known to influence both understory communities, edaphic properties and microbial assemblages (Hortal et al., 2015) and may thus influence nematode communities through shifts in these.

*Dasiphora fruticosa* is a common woody shrub in the alpine meadows of the Tibetan Plateau that plays an important role in preventing grassland degradation (Qian et al., 2021). Wang et al. (2019) showed that *D. fruticosa* affects bacterial and fungal richness and through this influences soil nematode richness. *Ligularia virgaurea* is another common dominant perennial plant in the same ecosystem but it affects the ecosystem rather differently. Specifically, *L. virguarea* produces allelochemical compounds that affect the germination and growth of neighboring plants (Wu et al., 2011; Zhang et al., 2011) as well as soil physicochemical properties and microbial communities (Kalemba and Kunicka, 2003; Shi et al., 2011). Although the effect of allelopathic and facilitative plants on soil biota has been explored by other studies (Tewksbury and Lloyd, 2001; Rodríguez-Echeverría et al., 2013; Jabran et al., 2015), few studies have directly contrasted their direct and indirect effects on soil nematode communities.

In this study, we compared the direct and indirect pathways through which *Ligularia virgaurea* and *Dasiphora fruticosa* influence understory plant and belowground communities in an alpine meadow on the Tibetan plateau. We used high-throughput sequencing to assess richness and composition of soil microbes and nematodes. Morphological identification of nematodes is time-consuming and requires excellent taxonomic skills, and is generally limited by incomplete description of local nematode species (Morise et al., 2012). High-throughput sequencing technology has been shown to overcome these weaknesses (Kerfahi et al., 2016; Geisen et al., 2018; Wilschut et al., 2019). Compared with traditional methods, molecular methods for studying nematode communities have potential advantages in terms of resolution, yield, cost, and time (Bongiorno et al., 2019). We applied piecewise structural equation modelling to assess the direct and indirect effects of the two contrasting dominant plants on nematode community composition through changes in the soil properties, understory plant community and soil microbial communities.

We hypothesized that: (i) *Ligularia virgaurea* decreases understory plant, bacteria and nematode richness (Ade et al., 2021), and increases fungal richness (Shi et al., 2011), which both directly and indirectly affect soil nematode assemblages and (ii) *Dasiphora fruticosa* facilitates understory plants thereby increases resource availability, soil microbial and nematode richness, and influences soil nematode communities mainly indirectly through biotic (understory composition and microbial communities) and abiotic (soil properties) factors (Wang et al., 2018).

## Materials and methods

### Study site

The study site is a relatively flat alpine meadow of the Gansu Gannan Grassland Ecosystem National Observation and Research Station of Lanzhou University (Azi Branch Station) in Maqu (33°40′N, 101°51′E), Gannan Tibetan Autonomous Prefecture on the eastern edge of the Tibetan Plateau, Gansu Province. Azi Branch Station is located 3,500 m above sea level. The annual precipitation is 620 mm, and the rain falls mainly during the short, cool summer. There are approximately 2,580 h of cloud-free solar radiation annually (Wang et al., 2018). The study site is a typical sub-alpine meadow dominated by a few shrubs, including the shrubs, and annual herbaceous plants.

### Experimental design

In June 2016, a 30 m × 50 m grazed site with a healthy population of both *Dasiphora fruticosa* (> 200 individuals) and *Ligularia virgaurea* (> 100 individuals) was selected for the study. Five *D. fruticosa* and five *L. virgaurea*, all with similar crown sizes, were randomly picked for further investigation. The crown size for all plants sampled was around 1 m in diameter. To fit the sampling squares well within the crown coverage while avoiding the plant stem, we used a 30 × 30 cm square for understory vegetation surveys and collection of soil samples. Additionally, we established five 30 cm × 30 cm plots randomly in areas without *D. fruticosa* and *L. virgaurea* (>1 m from existing *D. fruticosa* and *L. virgaurea* canopy edges) to act as controls.

### Soil sampling and vegetation survey

In August 2016, at the end of the growing season, we recorded the species and number of plants within the 30 cm × 30 cm quadrate. Following the vegetation survey, we harvested all understory plant species, dried them at 65°C for 3 days, and calculated plant biomass. We also collected three soil cores from the centre of each plot using a soil auger (4 cm diameter, 20 cm depth), removed large stones and then hand-mixed the soil to form a composite sample in plastic bags. Soils were kept at 4°C until processing. A subsample of the soil from the mixed sample was transferred to a sterile 15 ml centrifuge tube and stored at −80°C for molecular analyses.

### Soil properties

Soil water content was measured by drying 30 g of soil for 72 h at 105°C. The remaining soil was air-dried, avoiding direct sunlight following the removal of gravel and plant residues by hand, and then sieved through a 100 mesh (0.15 mm). Soil pH was measured in a 1:2.5 soil: deionized water slurry using a pH meter (PHSJ-3F, Shanghai INESA Scientific Instrument Co., Ltd., China). Soil organic matter was measured following the dichromate oxidation procedure (Kalembasa and Jenkinson, 1973). Soil total nitrogen and phosphorus were estimated following digestion by concentrated H_2_SO_4_, and measured by semi-micro Kjeldahl and Mo-Sb antispetrophotography, respectively, using an auto chemistry analyzer (SmartChem 200, AMS Alliance) (Hendershot, 1985; Baillie, 1990). Soil ammonium and nitrate nitrogen were measured from 2 M KCl extracts using the auto chemistry analyzer (SmartChem 200, AMS Alliance) (Cui et al., 2022).

### Soil microbial identification

Genomic DNA was extracted from 0.25 g well-mixed soil according to the method on the DNeasy PowerSoil Kit (QIAGEN) (MoBio Laboratories, Carlsbad, CA, United States). Then we measured the DNA concentration using NanoDrop and performed 1% agarose gel electrophoresis on the DNA sample to ensure that the DNA samples could be used in the subsequent analyses. The DNA samples were sequenced using Illumina Miseq PE300 High-Throughput Sequencing. The primer pairs 341F (CCTACGGGNGGCWGCAG) and 785R (GACTACHVGGGTATCTAATCC) with barcodes were used for amplifying bacterial V3-V4 fragments (Klindworth et al., 2013). ITS1F (GATTGAATGGCTTAGAGG) and ITS2R (CTGCGTTCTTCATCGAT) with barcodes were used for amplifying fungal partial rDNA and ITS fragments (McGuire et al., 2013). The primers included Illumina adapters with the reverse primers also having an error-correcting 12-bp barcode unique to each sample to permit multiplexing of samples (Barberan et al., 2015). We used QIIME for quality-filtering (Caporaso et al., 2010). To remove the interference sequence, we (1) split the sequence into samples according to the barcode and removed the barcode, (2) deduplicated the double-end sequences by the “Trimmomatic” software: removed bases with a tail quality value lower than 25; a 50 bp sliding window was set, with a 1 bp step, and the average base quality in the window was not less than 25; sequences less than 100 bp were removed, and (3) connected high-quality double-end sequences, with a minimum overlap region of 10 bp and a maximum mismatch rate of 0.2, then removed sequences containing ambiguous base N by the “flash” software. The remaining sequences were clustered into OTUs using the uparse software^1^ according to their similarity. We used 97% similarity for phylogentic alignments, which generally represent microbial taxonomy at the species level. Bacterial phylogeny was assigned using the RDP database,^2^ and fungal ITS were compared with the Unite database^3^ We used the RDP classifier to compare the OTU representative sequence with the corresponding database to obtain the OTU species information, at a confidence threshold of 80%.

### Soil nematode identification

Nematode DNA extraction was consistent with that described above, with nematode sequences amplified from the genomic DNA. While using DNA extracted from 0.25 g soil sub-samples should be interpreted with cautions, recent papers have shown that insights can be provided using this approach (Baidoo et al., 2017; Sikder et al., 2020; Wu et al., 2021). Specifically, the approach appears robust in providing a fingerprint of nematode communities, including DNA from species that are present in the system at large but are not present in the sub-sample itself (i.e., extracellular nematode DNA) (Peham et al., 2017). In order to improve amplification efficiency, we repeated DNA amplification before sequencing. The primers NemF and 18Sr2b (Sikder et al., 2020) were used in a pre-amplification step followed by amplification with primers NF1 and 18Sr2b in a semi-nested procedure (Sapkota and Nicolaisen, 2015) (Supplementary Table S1). NF1 and 18Sr2b were tag encoded using the forward primer 5 -CGTATCGCCTCCCTCGCGCCATCAG-MID-NF1–3 and the reverse primer 5 -CTATGCGCCTTGCCAGCCCGCTCAG-18Sr2b-3 (Sapkota and Nicolaisen, 2015). Reactions contained 12.5 μL of 2 × Taq PCR mixture with loading dye reaction buffer (GenStar), 2.5 μL each of forward primer and reverse primer, 1 μL of DNA template, and 6.5 μL ddH_2_O in a final volume of 25 μL. Amplification with NemF and 18Sr2b used an initial DNA denaturation step of 94°C for 5 min, followed by 20 cycles at 94°C for 30 s, 53°C for 30 s, 72°C for 1 min and a final elongation at 72°C for 10 min (Sapkota and Nicolaisen, 2015). After amplification, DNA samples were subjected to 1% agarose gel electrophoresis to check whether they can be used for Illumina Miseq PE300 High-Throughput sequencing. Quality-filtering was consistent with soil microbial methods. Valid sequences without chimeras were subsequently clustered into different OTUs (Operational Taxonomic Units) by uparse (see text footnote 1) according to their similarities, and a 99% similarity level was selected here. The RDP classifier was used to compare the OTU representative sequence with the Silva 18S (version 123) database to obtain OTU species information. The confidence threshold used by the RDP classifier to compare species databases was 80%.

### Data analyses

We calculated OTUs richness for soil microbes and nematodes. For plants, we calculated understory species richness. We used Levene’s test to test the homogeneity of variance and Shapiro–Wilk test to test the normality of data. If the data showed normal distribution and homogeneity of variance, nematode richness, soil variables, microbial richness and understory species richness and biomass were assessed for differences between treatments using one-way analysis of variance (ANOVA) followed by Tukey HSD *post hoc* tests when main effects were observed. If the data did not conform to the assumptions of normal distribution and variance homogeneity, we used permutation analysis of variance tests followed by Dunn’s *post hoc* comparison (if necessary).

Non-metric multidimensional scaling (NMDS) based on the Bray–Curtis dissimilarity index was used to visualize differences in nematode, plant, bacterial and fungal communities among treatments. Non-parametric multivariate analysis of variance (NPMANOVA) with 9,999 permutations was used to test for differences in plant, soil microbe, and soil nematode community composition between plant treatments.

Our small sample size limits the capacity to fit complex models; hence, we carried out piecewise structural equation modelling (piecewise SEM) to explore the possible underlying mechanisms behind the effects of plants on soil nematodes ranging from direct effects to indirect effects induced through changes in edaphic, understory and microbial communities. The goodness of piecewise SEM was evaluated by Shipley’s test of d-separation through Fisher’s C statistic, and Fisher’s C was statistically nonsignificant (*p* > 0.05) means a good model fit (Shipley, 2009). Soil physico-chemical properties were standardized using min-max normalization prior to model fitting. The soil biota and plant matrix were subjected to a Principal Component Analysis (PCA) with Hellinger-transformed to extract PC1, which summarized the variation in community composition.

We used an *a priori* model as a framework for the piecewise-SEM analysis (Veen et al., 2010) following these premises: (1) soil nematode richness and community composition can be affected by soil physicochemical properties, understory plants (richness and community composition), soil microbes (richness and community composition), and dominant plant species (Yeates et al., 1993; Wang et al., 2018), (2) understory plants and soil microbial richness and community composition can be affected by soil physicochemical properties, and dominant plant species (Hortal et al., 2015), and (3) soil physicochemical properties can be affected by dominant plant (Wang et al., 2018).

All data were analyzed using R software, version 4.1.3 (R Core Team). The variance homogeneity was checked using the ‘car’ package (Cui et al., 2022), the permutation test was conducted with the ‘lmPerm’ package (Wang et al., 2018), the Dunn’s test was conducted with the ‘pgirmess’ package (Khodaei and Long, 2019), PCA and NMDS were conducted with the ‘vegan’ package (Benito et al., 2018), piecewise SEM was calculated with ‘piecewiseSEM’ package (Cui et al., 2022). The figures were plotted using the ‘ggplot2’ package (Yang et al., 2021).

## Results

### Understory vegetation

**Figure 1:**
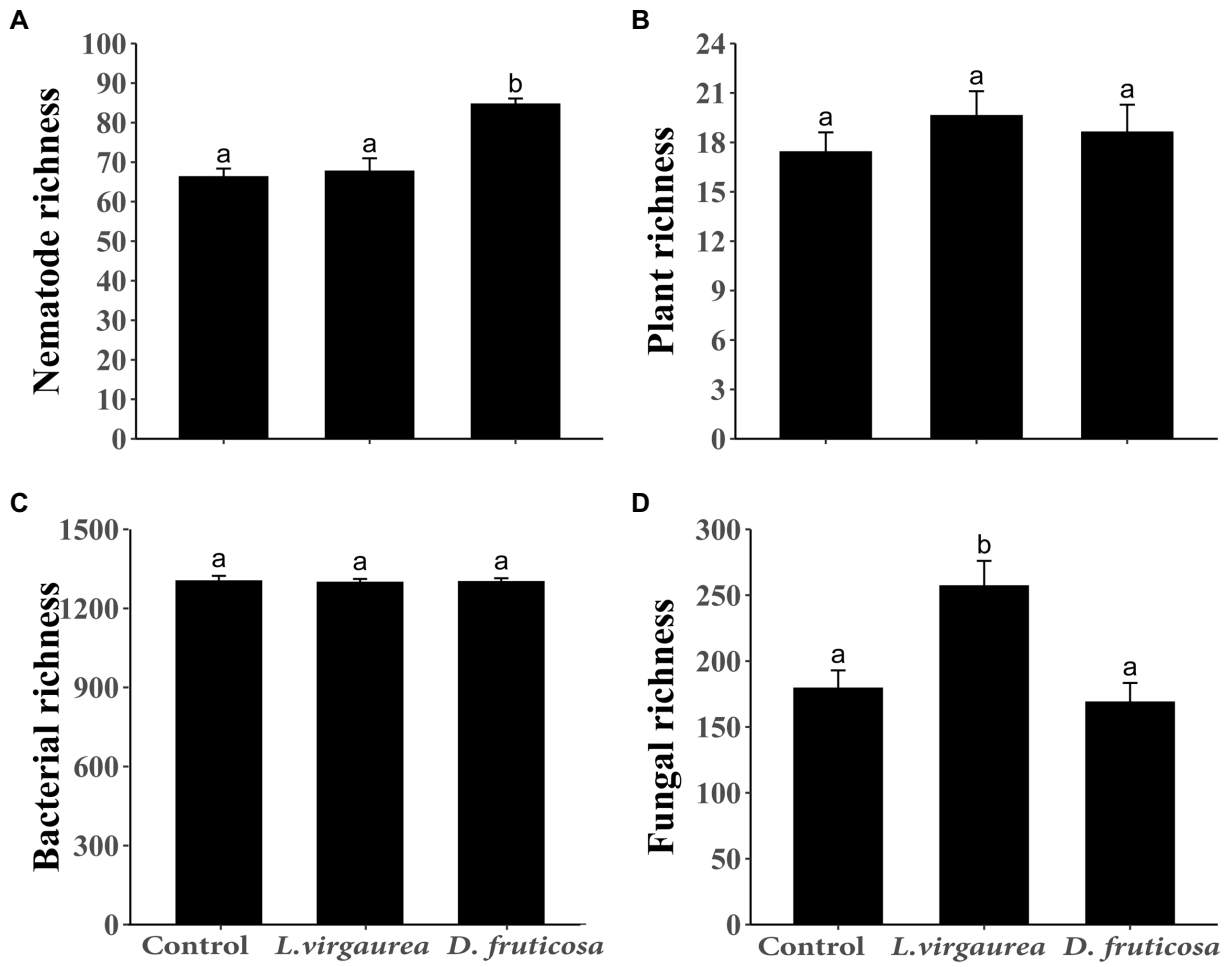
Mean (± SE) **(A)** nematode OTU richness, **(B)** understory plant richness, **(C)** bacterial OTU richness, and **(D)** fungi OTU richness across the treatments. Different letters indicate significant differences among treatments (*p* < 0.05) as determined by Tukey HSD test.

**Figure 2:**
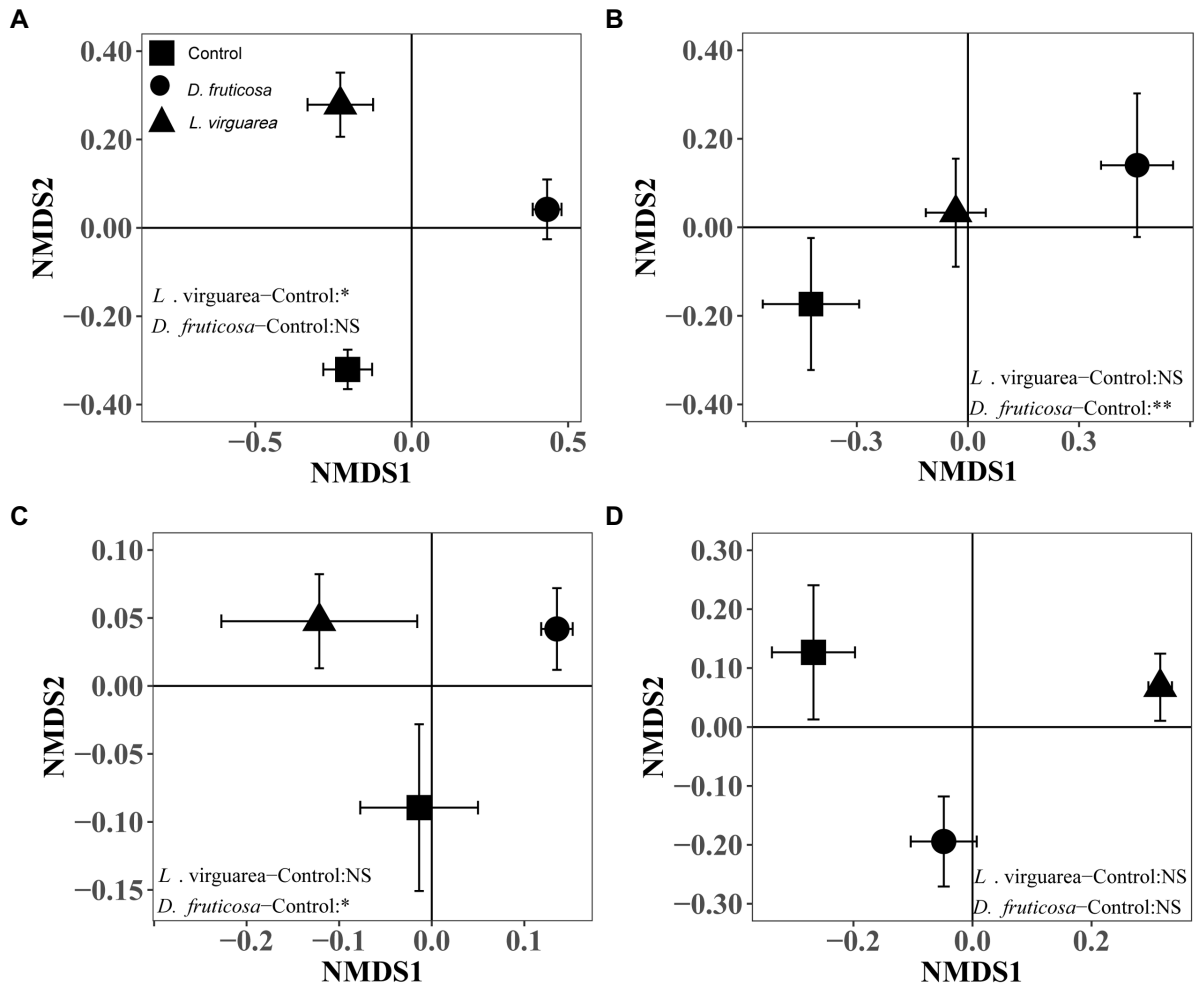
Community composition (Mean ± SE) of **(A)** nematodes, **(B)** understory plants, **(C)** bacteria, and **(D)** fungi associated with each plant treatment (i.e., *L. virgaurea* and *D. fruticosa*) based on non-metric multidimensional scaling (NMDS) using Bray–Curtis similarity index (stress<0.2). Significant results of one-way non-parametric multivariate analysis of variance (NPMANOVA) using Bray–Curtis similarity index are indicated on the right or left bottom part of each graph. ^**^: *p* < 0.01, ^*^: *p* < 0.05, (.): *p* < 0.1, NS: *p* > 0.05.

The presence of *L. virgaurea* and *D. fruticosa* had no effect on understory plant richness (Figure 1B), but plant biomass was significantly affected (Supplementary Figure S3), with greater biomass under *L. virgaurea* than *D. fruticosa* (Supplementary Figure S3, *p* < 0.05). Specifically, the biomass of *Poa pachyantha* was greater under *D. fruticosa* than in the control treatment (Supplementary Table S2, *p* < 0.001), while the biomass of *Potentilla anserine* was lower under *D. fruticosa* than in the control treatment (Supplementary Table S2, *p* < 0.05). The abundance of *Elymus nutans* (*p* < 0.01) was greater under *L. virgaurea* than *D. fruticosa* and in the control treatment (Supplementary Table S3). Non-metric multidimensional scaling (NMDS) and non-parametric multivariate analysis of variance (NPMANOVA) results showed that understory plant community composition based on presence/absence data differed significantly between *D. fruticosa* and the control treatment (Figure 2B, *p* < 0.01), while there was no significant difference between *L. virgaurea* and the control treatment (*p* > 0.1).

### Soil physicochemical properties

Soil total nitrogen (*p* < 0.05), soil organic carbon (*p* < 0.05), and soil ammonium nitrogen (*p* < 0.01) were greater in the presence of *D. fruticosa* relative to the control treatment, while soil pH (*p* < 0.01) was lower. Soil ammonium nitrogen (*p* < 0.01) was greater in the presence of *L. virgaurea* relative to the control treatment (Table 1). Neither *D. fruticosa* nor *L. virgaurea* significantly changed soil water content, total phosphorus and nitrate (*p* > 0.05). There was no significant difference between *L. virgaurea* and *D. fruticosa* in soil physico-chemical properties.

**Table 1:** Effects of different plant types on soil physicochemical properties (Mean ± SE).

	Control	*L. virgaurea*	*D. fruticosa*	*p*-value
SWC	0.43 ± 0.02	0.44 ± 0.02	0.39 ± 0.01	0.241
TP	0.89 ± 0.05	0.89 ± 0.03	0.97 ± 0.01	0.177
**TN**	**11.14 ± 0.74a**	**11.91 ± 0.46ab**	**13.46 ± 0.51b**	**0.044**
**SOC**	**98.06 ± 9.00a**	**120.21 ± 7.37ab**	**132.06 ± 7.71b**	**0.033**
**pH**	**6.77 ± 0.04b**	**6.63 ± 0.05ab**	**6.47 ± 0.03a**	**0.001**
NO_3_^−^-N	2.96 ± 0.38	2.23 ± 0.12	2.99 ± 0.23	0.112
**NH**_**4**_^**+**^ **-N**	**0.11 ± 0.04a**	**0.42 ± 0.06b**	**0.40 ± 0.06b**	**0.002**

SWC, soil water content (g/g); TP, total phosphorus (mg/g); TN, total nitrogen (mg/g); SOC, soil carbon content (mg/g); NO_3_^−^-N, nitrate nitrogen (mg/g); NH_4_^+^-N, ammonium nitrogen (mg/g). Letters after each value indicate results of pair-wise comparisons. Different letters indicate significant differences among treatments (*p* < 0.05) as determined by Tukey HSD test. Bold values mean *p* < 0.05.

### Soil biota

For bacteria, we retained a total of 683,830 sequences after filtering and removing chimeras. The total number of bases was 313,003,151, and the average sequence length 457.72 at 97% similarity. For fungi, we retained a total of 657,833 sequences after filtering and removing chimeras. The total number of bases was 224,765,243, and the average sequence length 341.68 at 97% similarity. There was no difference in bacterial richness among the treatments (Figure 1C). Fungal richness was greater under *Ligularia virgaurea* (Figure 1D, *p* < 0.01), but not *D. fruticosa*, relative to the control (Figure 1D). Non-metric multidimensional scaling (NMDS) and non-parametric multivariate analysis of variance (NPMANOVA) results showed that bacterial community composition under *D. fruticosa* was significantly different from that under control (Figure 2C, *p* < 0.05). However, there was no significant difference in fungal community composition among dominant plant types and the control treatment (Figure 2D).

The three most relatively abundant bacterial phyla detected in this study were Proteobacteria, Acidobacteria and Verrucomicrobia (Supplementary Figure S2C). The presence of *D. fruticosa* was associated with an increased relative abundance of Bacteroidetes but decreased relative abundance of Planctomycetes (Supplementary Table S4). The presence of *L. virgaurea* was associated with an increased relative abundance of Proteobacteria but decreased relative abundance of Planctomycetes (Supplementary Table S4). The three most relatively abundant fungal phyla detected in this study were Basidiomycota, Ascomycota and Glomeromycota (Supplementary Figure S2D). There were no differences in the relative abundance of the dominant fungal phyla among the treatments (Supplementary Table S5).

For Eukarya, we retained a total of 4,143,079 sequences after filtering and removing chimeras. The total number of bases was 1,536,595,586, and the average sequence length 370.88 at 99% similarity. Following the classification of OTUs, we found that the average nematode content was 41.81%, with other metazoan and fungi making up the remaining 58.19% (Supplementary Figure S2A). We found a significant positive effect of *D. fruticosa* on nematode richness (Figure 1A, *p* < 0.001), while *L. virgaurea* had no effect on nematode richness (Figure 1A). By contrast, *L. virguarea* had a significant effect on nematode community composition, associated with a decrease in the proportion of Araeolaimida (Supplementary Figure S2B; Supplementary Table S6), whereas *Dasiphora fruticosa* had no effect of nematode communities composition (Figure 2A).

### Piecewise structural equation modelling

**Figure 3:**
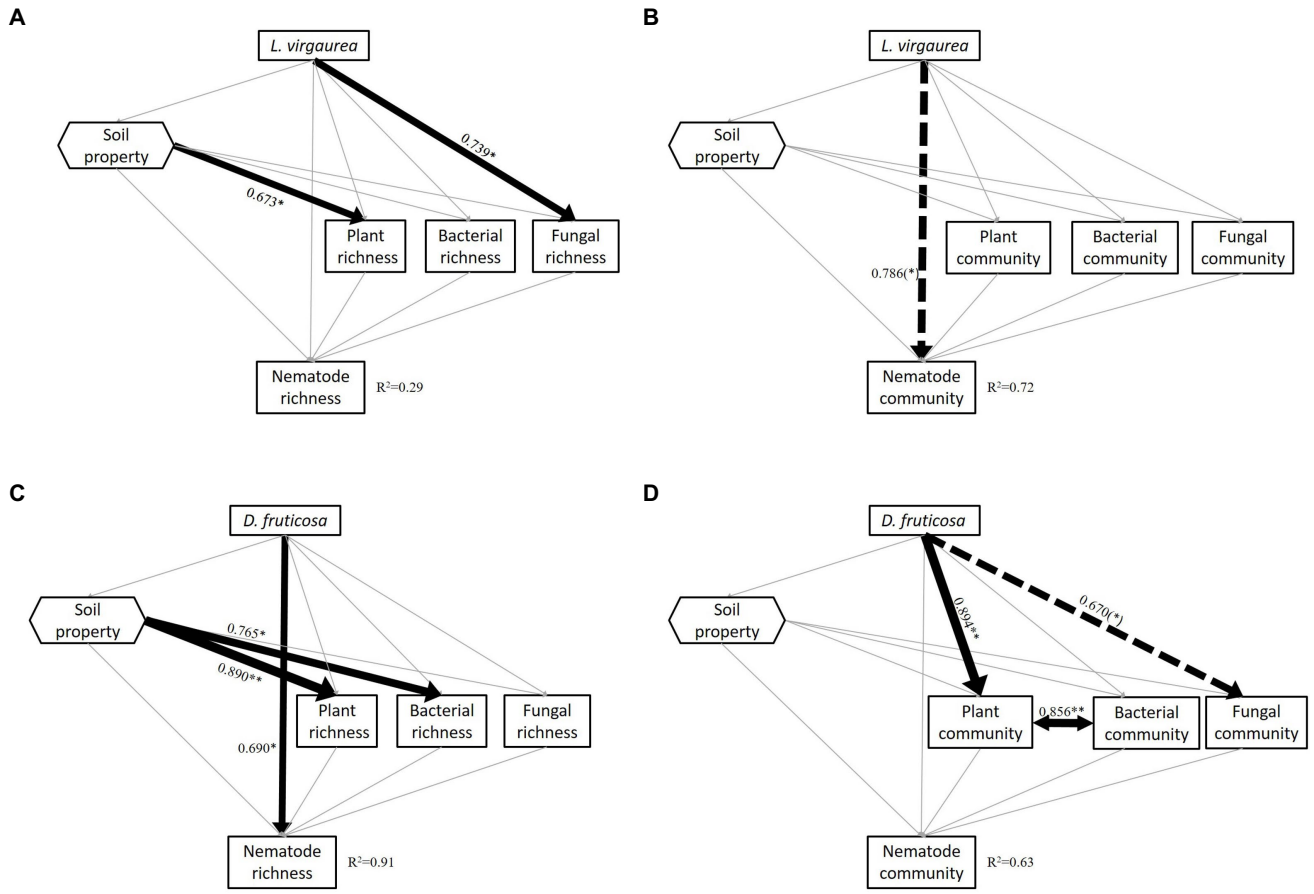
Results of the piecewise SEM analyses indicating direct and indirect effects of *L. virgaurea* on nematode **(A)** richness (Fisher’s C = 8.013, *p* = 0.237, AIC = 52.013, df = 6) and **(B)** community composition (Fisher’s C = 2.632, *p* = 0.621, AIC = 46.632, df = 4) and *D. fruticosa* on nematode **(C)** richness (Fisher’s C = 5.699, *p* = 0.458, AIC = 49.699, df = 6) and **(D)** community composition (PC1) (Fisher’s C = 3.115, *p* = 0.794, AIC = 47.115, df = 6). Square boxes display variables included in the model: Plant richness, understory plant richness; Plant community, understory plant community composition (PC1); Bacteria community, bacterial community composition (PC1); Fungi community, fungal community composition (PC1); Nematode community, nematode community composition (PC1). Black line means positive correlation and red line means negative correlation; grey line mean no significant correlation. Solid arrows indicate significant effects (*p* < 0.05), and dashed arrows indicate marginally significant effects (*p* < 0.1). Arrow width corresponds directly to the standardized path coefficient. *R*^2^ values associated with response variables indicate the proportion of explained variation by relationships with other variables. Values associated with solid arrows and dashed arrows represent standardized path coefficients.

The piecewise SEM model (*n* = 10) assessing the effects of *L. virgaurea* on nematode richness explained 29% of the variation. *L. virgaurea* had no effect on nematode richness. Although *L. virgaurea* had a direct and positive effect on soil fungal richness, there was no significant relationship between nematode richness and fungal richness (Figure 3A). The piecewise SEM model (*n* = 10) assessing the effect of *L. virguarea* on nematode community composition explained 72% of the variation. The presence of *L. virgaurea* had a direct effect on nematode community composition while no indirect effects were observed (Figure 3B).

The piecewise SEM model (*n* = 10) assessing the effects of *D. fruticosa* explained 91% of the variation in soil nematode richness. The presence of *D. fruticosa* had a direct positive effect on nematode richness with no indirect effects observed (Figure 3C). The piecewise SEM (*n* = 10) assessing the effects of *D. fruticosa* explained 63% of the variation of nematode community composition, with no effect of *D. fruticosa* on soil nematode community composition. There was a strong correlation between the community of understory and bacteria under *D. fruticosa*. Although we found *D. fruticosa* had a significant direct effect on understory plant community composition and a marginally significant direct effect on soil fungal community composition, and an indirectly effect on soil bacterial community composition, there was no significant correlation between nematode and understory plant and soil microbial community composition (Figure 3D).

## Discussion

Our study provides new insights into the influences of dominant plants on belowground communities. We found that some aspects of understory composition, edaphic properties and microbial communities differed between the two dominant plants. Moreover, nematode richness was greater in soils underneath *D. fruticosa*, while nematode communities in soils under *L. virgaurea* differed in composition relative to dominant plant-free controls. The effects of *D. fruticosa* on nematode richness and *L. virguarea* on nematode community composition were, however, not linked to observed changes in the understory plant community, microbial communities or edaphic variables. Our study thus provides evidence that dominant plants can have contrasting effects on soil nematode communities that should be explored in more detail in future studies.

### The effects of *Ligularia virguarea* on understory plants and soil biota

Our results indicate that *L. virgaurea* increases fungal richness and contributes to shifts in understory plant (marginally) and nematode community composition. Understory plant biomass was significantly greater under *L. virgaurea* than *D. fruticosa*, possibly due to understory plants benefitting from reduced influence of grazing because herbivores avoid *L. virgaurea* more than *D. fruticosa* (Shi et al., 2011), which may also have contributed to changes in understory plant abundance by altering competitive interactions. For example, the abundance of *Elymus nutans* (Poaceae), a preferred resource of herbivores, was significantly higher under *L. virgaurea* than under *D. fruticosa* (Supplementary Table S3). The increase in plant biomass would result in more litter returned belowground, which might explain the effect on fungal richness given that fungi are capable of actively decomposing recalcitrant components in leaf litter (Osono, 2020). Moreover, *Ligularia virgaurea* has been shown to release large amounts of volatile allelopathic compounds (Wu et al., 2011; Zhang et al., 2011; Arjmand et al., 2013) and to influence soil fungal community composition (Shi et al., 2011) and host-specific fungal endophytes (Aschehoug et al., 2014). Monoterpenoids are the main allelopathic substances produced by *L. virgaurea*, accounting for 16.6% of the total volatiles, with other allelopathic compounds including phenolic, terpenoid, alkane and aromatic compounds (Ma et al., 2005), which can also affect soil organisms (Misra et al., 2023). While both *L. virgaurea* roots and leaves produce allelopathic compounds, and leaf-derived allelopathic substances may enter soil through rainwater leaching, the allelopathic effect of roots has been shown to be stronger than that of leaves (Ma et al., 2006; Wu et al., 2011).

Although *L. virgaurea* did not significantly affect the soil bacterial community in our study, we found significant shifts in the relative abundance of Proteobacteria, Planctomycetes and Parcubacteria. Similarly, Liang et al. (2021) found that *L. virgaurea* increased the relative abundance of Proteobacteria. This might have important effects on ecosystem function as Proteobacteria are known to play a key role in soil C, N and sulphur cycling (Kersters et al., 2006).

The direct effect of *L. virgaurea* on the composition of nematode communities may be due to the release of allelochemicals, given that soil nematodes vary widely in their sensitivity to environmental disturbances (Hu et al., 2015). In particular, we found a reduction in the relative abundance of Araeolaimida (Supplementary Table S6). Similarly, root exudates of the allelopathic plant *Lantana camara* L. (Verbinaceae) have been shown to cause mortality of *Meloidogyne javanica* juveniles (Shaukat et al., 2003) and marigold (*Tagetes patula*) can produce allelopathic compounds toxic to plant parasitic nematodes (Marahatta et al., 2012). While our study indicates that allelopathic plants can affect soil nematode communities, further work is required to verify how leachate from *L. virguarea* affects nematode communities and which compounds might be involved.

### The effects of *Dasiphora fruticosa* on understory plants and soil biota

Our results further indicate that *D. fruticosa* increases soil nutrients and affects understory plant and bacterial community composition, indicating changes in resources that may result in cascading bottom-up effects (Peschel et al., 2019). Accordingly, we found an increase in nematode species richness but no other cascading effects on community composition. The positive response of nematode richness to *D. fruticosa* is likely through changes in root exudates (Bais et al., 2006) and input of litter (Chen et al., 2007; Chauvin et al., 2015). Soil organic carbon, total nitrogen and ammonium content were significantly greater under *D. fruticosa* than the controls (Table 1), which conforms with the “fertile island” effect observed in other studies (He and Li, 2016; Cai et al., 2020). *Dasiphora fruticosa* has been shown to release acidic substances into the rhizosphere that can alter soil pH and the solubility of nutrients (Gao et al., 2019), and can enhance nutrient availability by acquiring nutrients from deeper soil horizons that are ultimately returned to the upper soil horizons *via* litter or exudates (He and Li, 2016).

*Dasiphora fruticosa* had no significant effect on understory plant biomass, but influenced community composition, which might be caused by reduced grazing by herbivores and competition among plants. It is well known that shrub canopies can affect understorey community structure (Lozano et al., 2020). We found that under *D. fruticosa*, *Poa pachhyantha* biomass and abundance were considerably higher compared to the control treatment while *Potentilla anserine* biomass and abundance were significantly lower (Supplementary Tables S2 and S3). Similarly, Li et al. (2011) found that *D. fruticosa* facilitated some plant species in the herb layer, but this can result in increased competition for limited nutrients, soil water, light and space (Abd-ElGawad et al., 2020).

The presence of *D. fruticosa* also had a significant effect on bacterial community composition mediated predominantly by the effect of *D. fruticosa* on the understory plant community. Similarly, Hortal et al. (2013) found that the presence of the facilitative plant *Retama sphaerocarpa* increased the relative abundance of the gram-negative Proteobacteria and Bacteroidetes. A higher relative abundance of Proteobacteria and Bacteroidetes suggests that the soil communities are less disturbed and are considered a better resource for microbial grazers (Fierer et al., 2007).

We found that *D. fruticosa* had a direct effect on soil nematode richness. Wang et al. (2018) demonstrated a similar direct effect of *D. fruticosa* on soil nematode abundance at the same site. Dominant species can affect resource inputs (Bokhari, 1977; Stoler and Relyea, 2020) and stimulate the growth of microorganisms in the rhizosphere (Bardgett and Wardle, 2003); hence, the direct effect of *D. fruticosa* on soil nematode richness might be caused by increasing root activity, root leachate and plant litter. Our piecewise SEM indicated that *D. fruticosa* had a weak and marginally significant (*p* < 0.1) influence on soil fungal communities, but this was not supported by the NPMANOVA. This difference likely occurs because the path analysis accounts for other influences on the soil nematode community which the NPMANOVA does not. While the influence is relatively weak, this would be worthwhile exploring in more detail, including additional measurements of other variables that might drive community composition. In addition, our results did not indicate bottom-up cascade effects on the soil food web; however, a larger sample size is required to verify this finding. Future experiments should test the influence of *L. virguarea* and *D. fruticosa* on the complete soil food web more rigorously.

## Conclusion

Our study revealed that dominant plants with contrasting functional characteristics have markedly different impacts on soil nematode communities. The facilitative plant *D. fruticosa* increased soil nutrients and decreased soil pH, and had a positive effect on nematode richness. *Dasiphora fruticosa* also influenced understory plant and soil bacterial communities but had no significant effect on nematode community composition. The allelopathic plant *L. virguarea* increased soil ammonium and fungal richness, and influenced nematode community composition, largely due to a reduction in the relative abundance of Araeolaimids. Despite both plants affecting other ecosystem properties, our piecewise SEM revealed that the effects on nematode richness and community composition were unrelated to the influences on soil biotic and abiotic properties. Our study highlights the importance of dominant plants in determining soil community diversity and provides new insight to disentangle the complex above- and below-ground relationship.

## Data availability statement

The original contributions presented in the study are included in the article/ Supplementary material, further inquiries can be directed to the corresponding authors.

## Author contributions

HS, ZL, HC, JC, SC, HG, XY, YW, JW, KL, SX, LA, and UN contributed to the study conception and design. Material preparation, data collection and analysis were performed by HS, SX, XY, ZL, JC, HC, YW, JW, KL, SC, HG, LA, and UN. The first draft of the manuscript was written by HS. All authors contributed to the article and approved the submitted version.

## Funding

This work was supported by National Natural Science Foundation of China (projects 41830321, 32071532 and 31870412), the “111 Project” (BP0719040), the Natural Science Foundation of Gansu Province (22JR5RG564), and the Second Tibetan Plateau Scientific Expedition and Research Program (2019QZKK0302). There was a preprint called “Direct and Indirect Effects on Soil Nematode Communities Differ Between Facilitative and Allelopathic Plants,” the current version has been significantly revised.

## Conflict of interest

The authors declare that the research was conducted in the absence of any commercial or financial relationships that could be construed as a potential conflict of interest.

## Publisher’s note

All claims expressed in this article are solely those of the authors and do not necessarily represent those of their affiliated organizations, or those of the publisher, the editors and the reviewers. Any product that may be evaluated in this article, or claim that may be made by its manufacturer, is not guaranteed or endorsed by the publisher.

## Footnotes

^1^ http://www.drive5.com/usearch/manual/uparseotu_algo.html

^2^ http://rdp.cme.msu.edu/misc/resources.jsp

^3^ https://unite.ut.ee/index.php
