# Supplementary material for: Contrasting influences of two dominant plants, Dasiphora fruticosa and Ligularia virguarea, on aboveground and belowground communities in an alpine meadow
Source: Front Microbiol. 2023 Apr 14;14:1118789. doi: 10.3389/fmicb.2023.1118789 (PMC10140320; doi:10.3389/fmicb.2023.1118789)
Supplement: Supplementary file 1 [file Data_sheet_1.docx]

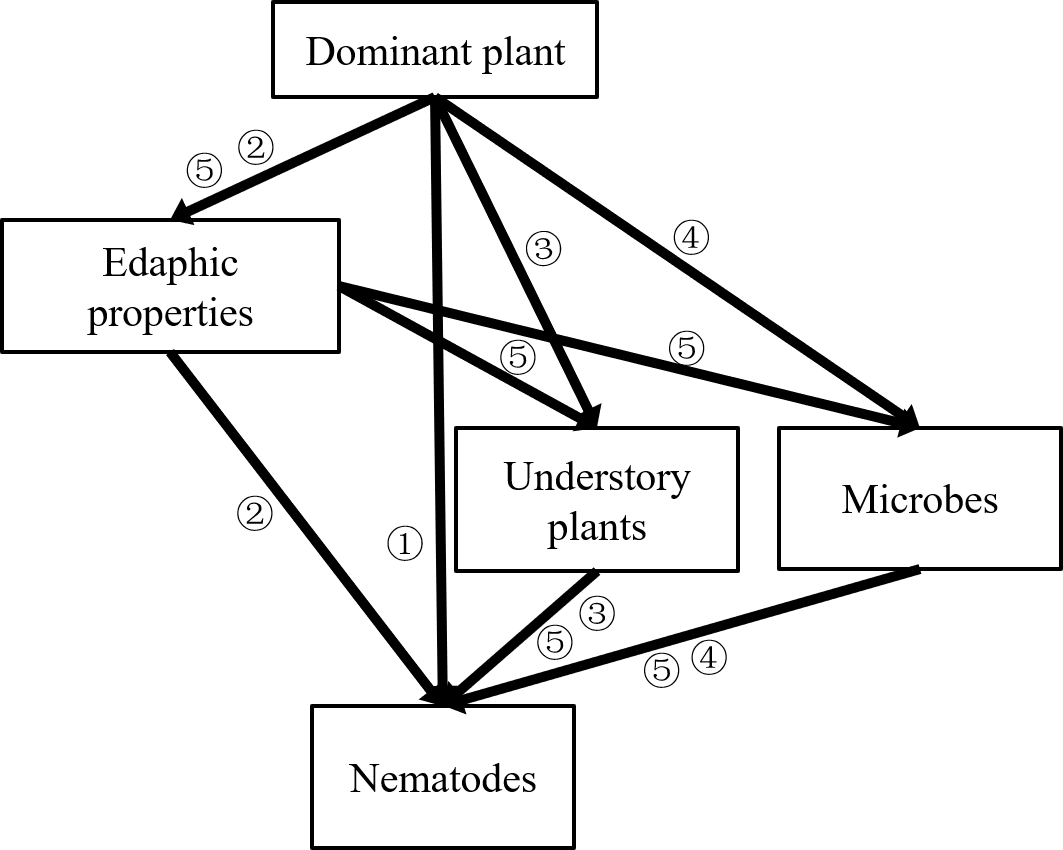


**Figure S1** A priory model assessing direct and indirect effects of dominant plants on soil nematodes. Pathway 1: tests for direct effects of the dominant plant on nematodes; pathway 2: tests indirect effects on nematode via changes in soil physicochemical properties; pathway 3: tests for indirect effects on nematodes via changes in understory plant communities; pathway 4: tests for indirect effects on nematodes via changes in soil microbial communities; pathway 5: tests for indirect effect on nematodes via changes in soil physicochemical properties, then shifts in understory plant and microbial communities.


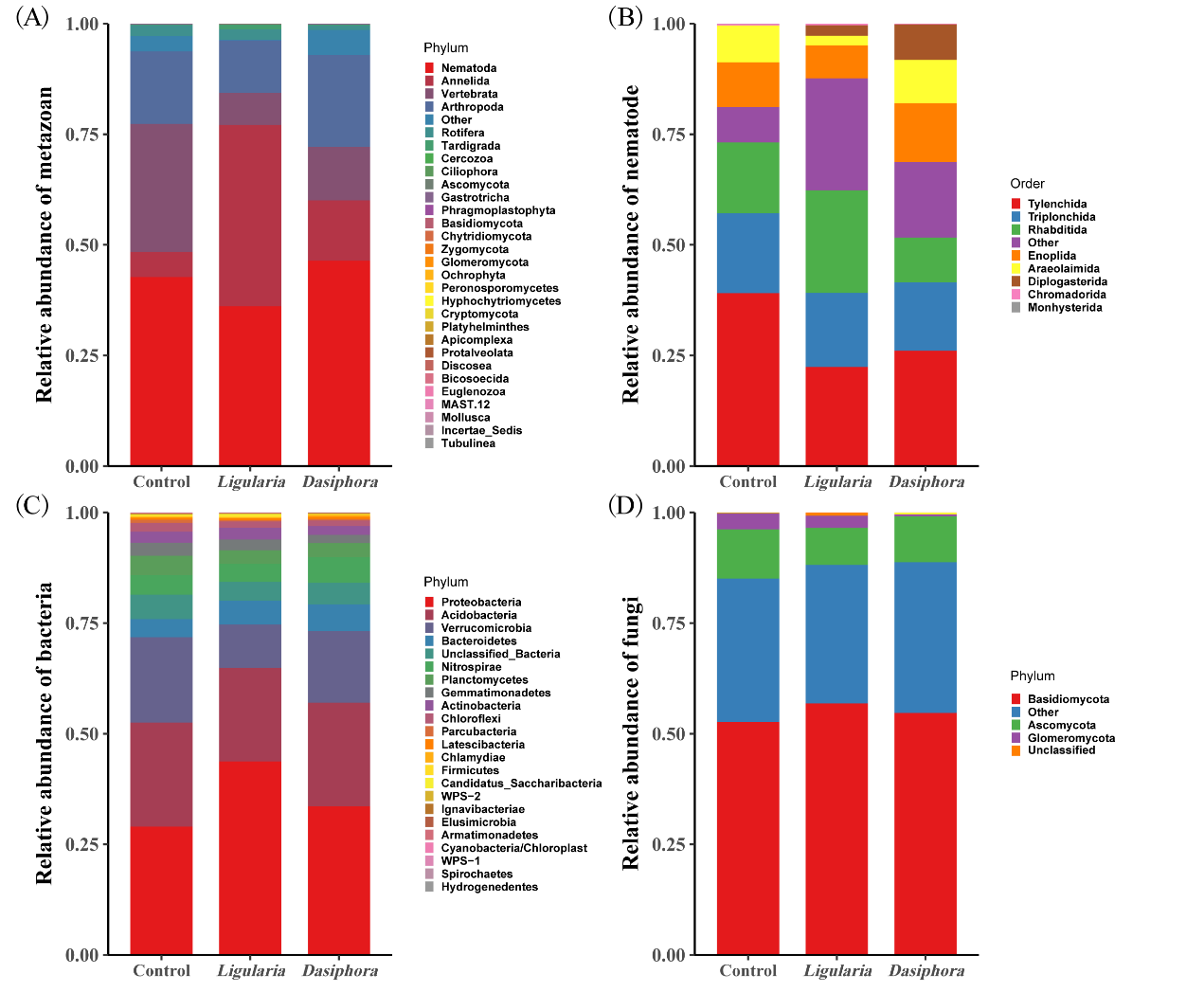


**Figure S2** Effects of dominant plants on the relative abundance of the phyla of (A) metazoans, (C) bacteria and (D) fungi, and (B) nematode orders.


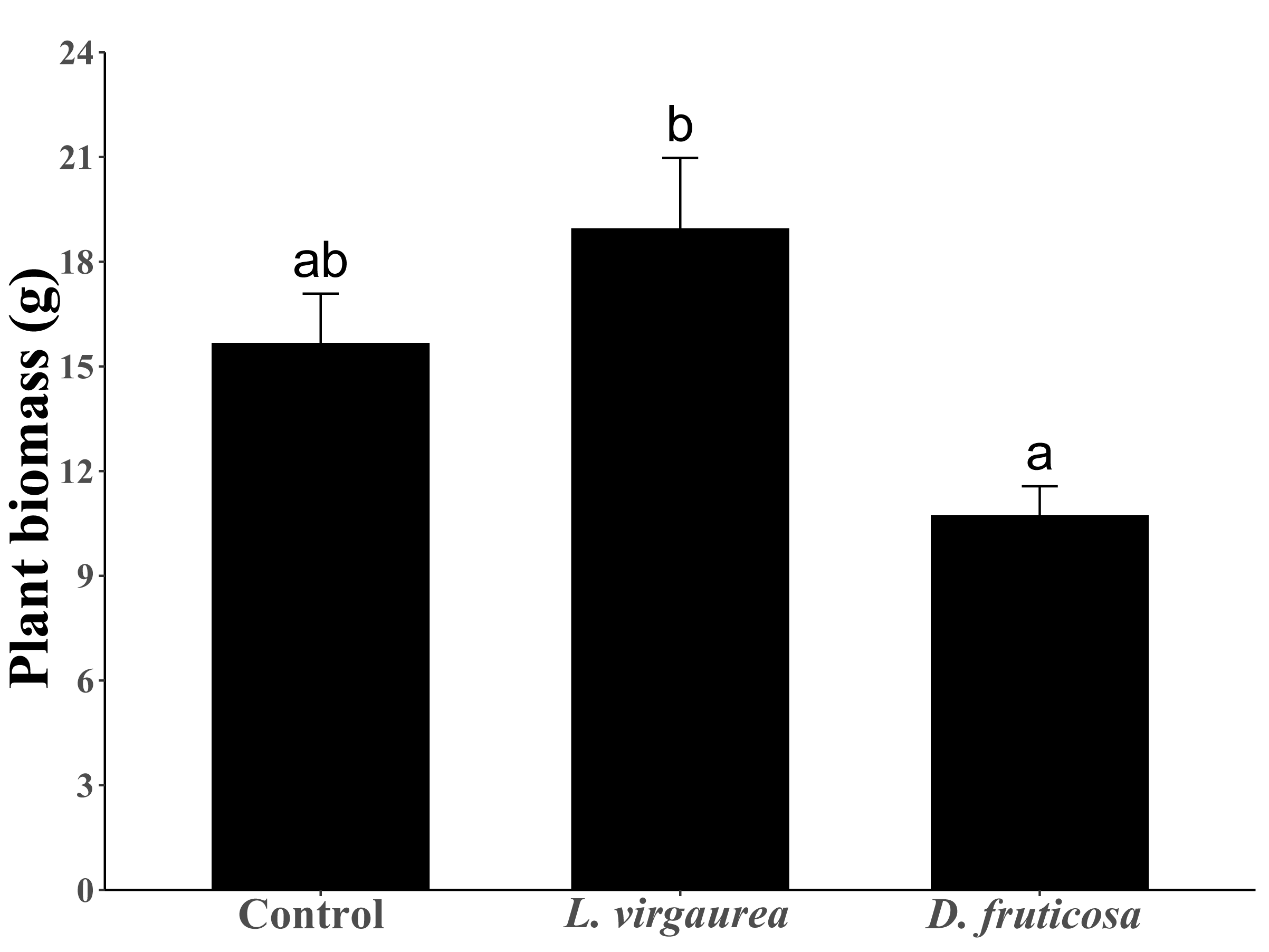


**Figure S3** Effects of dominant plants on the understory plant biomass (Mean ± SE). Different letters indicate significant differences among treatments (P < 0.05) as determined by Tukey HSD test.

Table S1 Nematode primers used in this study.

| Primer | 5’-3’ sequence | Reference | |
| --- | --- | --- | --- |
| NemF | GGGGAAGTATGGTTGCAAA | | (Sapkota and Nicolaisen, 2015) |
| NF1 | GGTGGTGCATGGCCGTTCTTAGTT | | (Porazinska et al., 2009) |
| 18Sr2b | TACAAAGGGCAGGGACGTAAT | | (Porazinska et al., 2009) |

Table S2 Biomass (g) of understory plant (dominant 10 species) under different treatments (Mean ± SE). Different letters indicate significant differences among treatments (P < 0.05) as determined by Tukey HSD test. Bold values mean P < 0.05.

|  | Control | *L. virgaurea* | *D. fruticosa* | P-value |
| --- | --- | --- | --- | --- |
| *Elymus nutans* | 2.55±0.73 | 4.67±1.56 | 2.04±0.8 | 0.238 |
| *Carex atrofusca* | 2.93±1.42 | 2.01±0.5 | 0.6±0.2 | 0.158 |
| ***Potentilla anserina*** | **2.94±1.19b** | **1.7±0.44b** | **0.28±0.11a** | **0.043** |
| *Scirpus pumilus* | 1.04±0.32a | 2.05±0.72a | 0.35±0.09a | 0.068 |
| ***Poa pachyantha*** | **0.11±0.06a** | **0.31±0.11ab** | **2.42±0.48b** | **<0.001** |
| *Pleurospermum camtschaticum* | 0.04±0.04 | 1.53±0.87 | 0.41±0.38 | 0.139 |
| *Leontopodium souliei* | 0.41±0.2 | 0.64±0.49 | 0.61±0.53 | 0.964 |
| *Kobresia capillifolia* | 0.16±0.1 | 0.42±0.26 | 0.8±0.2 | 0.106 |
| ***Potentilla saundersiana*** | **1.04±0.54b** | **0.06±0.06a** | **0.04±0.04a** | **0.036** |
| *Anemone rivularis* | 0.11±0.11 | 0.99±0.98 | 0±0 | 0.5 |

Table S3 Abundance of understory plant (dominant 10 species) under different treatments (Mean ± SE). Different letters indicate significant differences among treatments (P < 0.05) as determined by Tukey HSD test. Bold values mean P < 0.05.

|  | Control | *L. virgaurea* | *D. fruticosa* | P-value |
| --- | --- | --- | --- | --- |
| *Carex atrofusca* | 52±29.85 | 40.6±8.51 | 7.8±2.75 | 0.151 |
| ***Elymus nutans*** | **13±1.97a** | **32.4±6.93b** | **7.2±2.33a** | **0.004** |
| ***Potentilla anserina*** | **30.4±9.22b** | **15.2±2.71ab** | **3.4±1.12a** | **0.018** |
| *Scirpus pumilus* | 18.8±6.98 | 22.4±7.14 | 7±2.1 | 0.196 |
| *Leontopodium souliei* | 7.2±3.18 | 15±9.8 | 6.8±4.62 | 0.667 |
| ***Poa pachyantha*** | **1±0.45a** | **3±1.05ab** | **14.6±5.45b** | **0.014** |
| *Viola pseudo-bambusetorum* | 11.8±11.8 | 0.4±0.24 | 3.2±1.83 | 0.983 |
| *Anaphalis lactea* | 2±1.14 | 1.6±1.36 | 11±6.81 | 0.174 |
| *Potentilla saundersiana* | 13.2±7.56 | 0.6±0.6 | 0.2±0.2 | 0.056 |
| *Euphorbia altotibetica* | 2.6±2.6 | 5.4±2.64 | 4.4±2.5 | 0.783 |

Table S4 Relative proportion of bacteria (dominant 10 phyla) under different treatments (Mean ± SE). Different letters indicate significant differences among treatments (P < 0.05) as determined by Tukey HSD test. Bold values mean P < 0.05.

|  | Control | *L. virgaurea* | *D. fruticosa* | P-value |
| --- | --- | --- | --- | --- |
| **Proteobacteria** | **29.022±1.636a** | **43.75±5.869b** | **33.635±0.707ab** | **0.034** |
| Acidobacteria | 23.489±0.822 | 21.121±1.523 | 23.379±0.353 | 0.221 |
| Verrucomicrobia | 19.328±2.953 | 9.832±3.481 | 16.156±1.06 | 0.077 |
| **Bacteroidetes** | **4.07±0.369a** | **5.314±0.631ab** | **6.049±0.317b** | **0.031** |
| Nitrospirae | 4.422±0.122 | 4.127±1.035 | 5.831±0.391 | 0.143 |
| **Planctomycetes** | **4.392±0.234b** | **3.077±0.368a** | **3.186±0.193a** | **0.009** |
| Gemmatimonadetes | 2.895±0.32 | 2.421±0.388 | 1.856±0.182 | 0.098 |
| Actinobacteria | 2.538±0.236 | 2.631±0.466 | 1.992±0.102 | 0.318 |
| Chloroflexi | 2.009±0.394 | 1.44±0.182 | 1.256±0.076 | 0.086 |
| **Parcubacteria** | **0.687±0.13b** | **0.33±0.065a** | **0.371±0.046ab** | **0.027** |

Table S5 Relative proportion of fungi (dominant 4 phyla) under different treatments (Mean ± SE). Different letters indicate significant differences among treatments (P < 0.05) as determined by Tukey HSD test. Bold values mean P < 0.05.

|  | Control | *L. virgaurea* | *D. fruticosa* | P-value |
| --- | --- | --- | --- | --- |
| Basidiomycota | 51.707±16.041 | 55.865±12.287 | 53.665±11.632 | 0.976 |
| Ascomycota | 10.908±5.283 | 8.246±2.906 | 10.203±2.067 | 0.870 |
| Glomeromycota | 3.515±2.604 | 2.709±2.023 | 0.417±0.142 | 0.379 |
| Chytridiomycota | 0.097±7e-04 | 0.007±0.005 | 0.391±0.318 | 0.309 |

Table S6 Relative abundance of nematode (order) under different treatments (Mean ± SE). Different letters indicate significant differences among treatments (P < 0.05) as determined by Tukey HSD test. Bold values mean P < 0.05.

|  | Control | *L. virgaurea* | *D. fruticosa* | P-value |
| --- | --- | --- | --- | --- |
| Tylenchida | 32351.4±12247.487 | 12714±2915.386 | 20181.8±7053.446 | 0.288 |
| Triplonchida | 14971.6±4013.762 | 9507±1869.273 | 11937.6±2512.644 | 0.423 |
| Rhabditida | 13234.2±7093.684 | 13147±4366.362 | 7835.2±3373.983 | 0.771 |
| Enoplida | 8377±2524.658 | 4220±2439.705 | 10271±4714.561 | 0.361 |
| **Araeolaimida** | **6920.6±1850.014b** | **1230.2±410.961a** | **7582±2579.13b** | **0.037** |
| Diplogasterida | 0±0 | 1365.4±1183.46 | 6233±6015.329 | 0.318 |
| Chromadorida | 246.8±144.105 | 156±112.384 | 72.4±45.212 | 0.573 |
| Monhysterida | 72.6±59.499 | 33±17.539 | 37.8±20.814 | 0.726 |
| Other | 6603.2±2451.66 | 14369.4±4702.82 | 13239.4±7099.133 | 0.503 |

References

**Porazinska, D.L., Giblin-Davis, R.M., Faller, L., Farmerie, W., Kanzaki, N., Morris, K., Powers, T.O., Tucker, A.E., Sung, W., and Thomas, W.K.** (2009). Evaluating high-throughput sequencing as a method for metagenomic analysis of nematode diversity. Molecular ecology resources **9,** 1439-1450.

**Sapkota, R., and Nicolaisen, M.** (2015). High-throughput sequencing of nematode communities from total soil DNA extractions. BMC ecology **15,** 3.
